# Retraction: Emerging roles of the long non-coding RNA 01296/microRNA-143-3p/MSI2 axis in development of thyroid cancer

**DOI:** 10.1042/BSR-2018-2376_RET

**Published:** 2022-12-19

**Keywords:** AKT/STAT3 signaling pathway, Competing endogenous RNA, Long non-coding RNA 01296, microRNA-143-3p, Musashi 2, Thyroid cancer

This article is being retracted from *Bioscience Reports* at the request of the Editor-in-Chief and the Editorial Board following receipt of a notification from a reader alerting the Editorial Board to an apparent duplicate region within the si-NC and the miR-143-3p mimic + pcDNA-MSI2 panels of Figure 7H. Additional concerns exist over the western blots within the paper. The authors have been contacted with regards to the retraction and have not responded to the Journal's queries or the concerns raised. Given the extent of the issues raised, the Editorial Board stand by the decision to retract the article.

